# Posttraumatic Pancreatitis Four Days after Renal Injury with Massive Retroperitoneal Hematoma

**DOI:** 10.1155/2021/6693259

**Published:** 2021-05-07

**Authors:** Masamichi Kiriyama, Kei Jitsuiki, Ken-ichi Muramatsu, Hoshiko Furusawa, Soshi Moriya, Youichi Yanagawa

**Affiliations:** Department of Acute Critical Care Medicine, Shizuoka Hospital, Juntendo University, 1129 Nagaoka Izunokuni City Shizuoka, Japan 410-2295

## Abstract

A 25-year-old man accidentally fell from a cliff and hit his right flank on the ground while camping. Initially, he was able to barely walk, but he ultimately became unable to walk at all due to severe flank pain. He had no remarkable personal or family history and was a social drinker. Upon arrival, he showed clear consciousness but was in a hemorrhagic shock state. Enhanced computed tomography (CT) revealed extravasation of contrast medium from the injured right kidney with massive retroperitoneal hematoma. He underwent massive blood transfusion and tracheal intubation followed by renal embolization. His vital signs stabilized on hospital day 2, and he was extubated on day 3. On days 4 and 5, a blood examination revealed increased levels of amylase (360 and 904 IU/L, respectively). Enhanced CT on day 5 did not show signs of severe acute pancreatitis. The maximum amylase level was 1041 IU/L on day 6 and decreased day by day without deterioration of the severity of his acute pancreatitis. He was discharged on day 14. The subacute phase of posttraumatic acute pancreatitis in the present case may have been induced not by direct injury to the pancreas but by several causative factors, such as shock, increased pressure of the retroperitoneal space, or the release of inflammatory mediators from injured tissues or hematoma.

## 1. Introduction

Acute pancreatitis, an inflammatory disorder of the pancreas, is the leading cause of admission to the hospital for gastrointestinal disorders in many countries. Gallstones and alcohol misuse are long-established risk factors [1–3]. However, acute pancreatitis induced by traumatic injury is rare and difficult to diagnose.

The most evident findings of posttraumatic pancreatitis are blood, edema, and soft tissue infiltration of the anterior pararenal space [4]. The typical clinical triad of posttraumatic pancreatitis is upper abdominal pain, leukocytosis, and elevated serum amylase levels during the first 24 hours. Delayed diagnoses of posttraumatic pancreatitis are associated with high morbidity and mortality rates.

We herein report a case of complicating posttraumatic pancreatitis four days after renal injury with massive retroperitoneal hematoma and discuss the mechanism underlying the occurrence of posttraumatic pancreatitis in the present case.

## 2. Case Report

A 25-year-old man accidentally fell from a cliff and hit his right flank on the ground while camping. Initially, he was able to barely walk, but he ultimately became unable to walk at all due to severe flank pain. His colleague called an ambulance. He had no remarkable personal or family history and was a social drinker.

**Figure 1:**
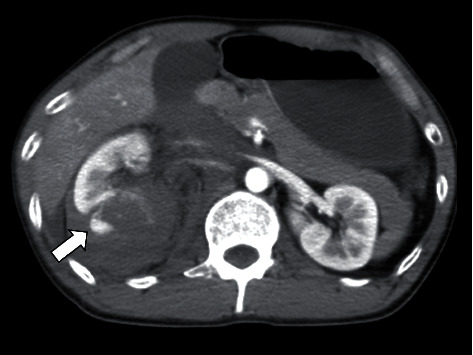
Enhanced abdominal computed tomography (CT) on arrival. CT showed extravasation of contrast medium from the injured right kidney. An accompanying massive retroperitoneal hematoma had pushed up the visceral organs into the ventral side.

**Figure 2:**
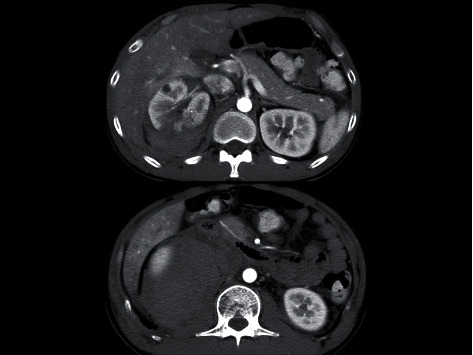
Enhanced abdominal computed tomography (CT) on hospital day 5. CT showed no exudative inflammation around the pancreas, which had been moved upward by the retroperitoneal hematoma.

Initially, he was transported to a local hospital, but computed tomography (CT) revealed right renal injury with massive retroperitoneal hematoma, so he was transported to our hospital by a physician-staffed helicopter 2 hours after the accident. Upon arrival, he showed clear consciousness but was in a hemorrhagic shock state. His vital signs were as follows: blood pressure, 84/50 mmHg; heart rate, 140 beats per minute; respiratory rate, 30 breaths per minute; percutaneous saturation; and 98% under 10 L/minute of oxygen via mask. He had marked right flank pain. A focused assessment with sonography in trauma was positive at Morrison's pouch. He urgently received 6 units of different-type blood transfusion, and his blood pressure temporarily increased. Enhanced CT revealed extravasation of contrast medium from the injured right kidney with massive retroperitoneal hematoma, which had pushed the visceral organs up into the ventral side (Figure 1). Blood test findings on arrival are shown in Table 1. On returning to the emergency room, he suffered hemorrhagic shock again and subsequently underwent repeated massive blood transfusion, including red blood cells, cryoprecipitate, fresh-frozen plasma, and platelets, and also received tracheal intubation and an indwelling intra-aortic balloon occlusion catheter (IABP) at zone I as a prophylactic measure against cardiac arrest. He was moved to the angio suite for interventional radiology and underwent transarterial selective renal artery embolization, after which he was admitted to the intensive care unit. His vital signs stabilized on hospital day 2. The IABP was removed without inflation. He received 12 units of red blood cell and fresh-frozen plasma, 20 units of platelets, and 4 units of cryoprecipitate within 24 hours. He was extubated on day 3 after initiating diuresis. CT on the same day revealed renal partial infarction without pseudoaneurysmal formation or urinoma. On days 4 and 5, a blood examination revealed increased levels of amylase (360 and 904 IU/L, respectively) with new additional epigastralgia. Enhanced CT on day 5 did not show exudative inflammation around the pancreas, which was still shifted upward by the retroperitoneal hematoma (Figure 2). Amylase isozyme patterns on day 5 identified the pancreas types, and the lipase level was 3061 IU/L. After receiving a diagnosis of mild-grade acute pancreatitis based on CT findings [5], he abstained from food on days 5 and 6. As the severity of acute pancreatitis was mild, he began to eat again from day 7. The maximum amylase level was 1041 IU/L on day 6 and decreased day by day without deterioration of the severity of his acute pancreatitis (Table 2). Magnetic resonance cholangiopancreatography on day 12 showed no injury to the main pancreatic duct. As he was able to eat and walk, he was discharged on day 14.

## 3. Discussion

We encountered a case of posttraumatic acute pancreatitis that occurred four days after renal injury with massive retroperitoneal hematoma. This case had no gallbladder stones or alcoholism, so these factors were not the cause of the acute pancreatitis. Drug-induced acute pancreatitis is a rare disease, and the drugs used in the present case during the increase of the amylase level are not listed as major causative drugs of acute pancreatitis [6, 7]. In cases of acute pancreatitis induced directly by trauma, the changes associated with direct traumatic pancreatitis may not be visualized within the early hours following the traumatic event, as they are time-dependent. However, CT findings even on day 5 in the present case failed to indicate any significant changes in the pancreas. An increase in amylase levels beyond the normal range is also usually recognized within six hours from the injury, but such findings in the present case were not recognized until four days later [4, 8, 9]. Accordingly, possibility of direct trauma to the pancreas as the cause of the posttraumatic acute pancreatitis in the present case was minimized.

Shock and pancreatitis are closely associated. Shock can be a sequela of severe pancreatitis, and systemic shock may induce pancreatitis [10]. The susceptibility of the pancreas to ischemia/reperfusion injury has been demonstrated in experimental studies as well as in clinical settings, such as cardiopulmonary bypass, hemorrhagic shock, and transplantation of the pancreas [11]. Oxygen free radicals, activation of polymorphonuclear leukocytes, failure of microvascular perfusion, cellular acidosis, and disturbance of intracellular homeostasis appear to be important factors/mechanisms in the pathogenesis of ischemia/reperfusion-induced acute pancreatitis [11]. The present case demonstrated prolonged hemorrhagic shock due to renal injury, so the shock may have been a causative factor of posttraumatic pancreatitis in the present case. There has been no concrete report indicating when shock inducing pancreatitis occurred. Warshaw and O'Hara reported that 13 patients with shock (3 septic, 2 hemorrhagic, 7 cardiogenic, and 1 induced intentionally for hypotensive anesthesia) had a normal serum amylase concentration during or shortly after the shock episode but developed hyperamylasemia over the following several days [12], while Malinoski et al. reported that elevated serum levels of pancreatic enzymes in patients in shock or who required a massive transfusion were independent predictors of organ failure [13]. In their study, the median times to reach an amylase level of 260 U/L or a lipase level of 120 U/L (both over twice the upper limit of the normal range) were 16 and 5.6 h, respectively. Accordingly, the hyperamylasemia in the present case may have shown a delayed onset if it had been induced by hemorrhagic shock.

Kotzampassi et al. investigated whether or not an increase in the retroperitoneal compartment pressure influences the pancreatic tissue blood flow through a simulation by infusing a colloid fluid into the retroperitoneum in swine [14]. As a result, the increase in the retroperitoneal compartment pressure was found to significantly increase the pancreatic interstitial pressure as well as reduce the pancreatic tissue blood flow. They therefore concluded that retroperitoneal fluid collection might contribute to or exacerbate pancreatic tissue ischemia, potentially resulting in the formation of acute pancreatitis. As the present case had massive retroperitoneal hematoma, this may have induced posttraumatic pancreatitis in the subacute phase.

We were unable to find any reports indicating direct associations between renal injury, renal infarction and/or retroperitoneal hematoma (red cell lysis and thrombin production), and acute pancreatitis. However, these factors can induce the release of a number of inflammatory mediators, such as cytokines, platelet-activating factor, endothelin, oxygen free radicals, and various chemokines [15–18]. Accordingly, such inflammatory mediators may spread into the pancreas and cause acute pancreatitis.

## 4. Conclusion

We herein report a case of posttraumatic pancreatitis that occurred four days after renal injury with massive retroperitoneal hematoma. The subacute phase of posttraumatic acute pancreatitis in the present case may have been induced not by direct injury to the pancreas but by several causative factors, such as shock, increased pressure of the retroperitoneal space, or the release of inflammatory mediators from injured tissues or hematoma.

## Figures and Tables

**Table 1:** Blood test findings on arrival.

pH	7.25
PCO_2_	23.3 mmHg
HCO_3_^−^	11.6 mmol/L
Base excess	-14.1 mmol/L
White blood cell	28.2 × 10^3^/mm^3^
Hemoglobin	11.1 g/dL
Platelets	25.0 × 10^4^/mm^3^
Total protein	5.8 g/dL
Total bilirubin	0.6 mg/dL
Aspartate aminotransferase	194 IU/L
Alanine aminotransferase	105 IU/L
Lactate dehydrogenase	392 IU/L
Alkaline phosphatase	188 IU/L
*γ*-Glutamyl transpeptidase	38 IU/L
Creatine kinase	580 IU/L
Amylase	42 IU/L
Glucose	68 mg/dL
Blood urea nitrogen	15.8 mg/dL
Creatinine	1.00 mg/dL
Sodium	142 mEq/L
Potassium	3.8 mEq/L
Prothrombin time-international normalized ratio	1.38
Activated partial thromboplastin time	27.1 sec
Fibrinogen	125 mg/dL
Fibrinogen degradation products	5.4 *μ*g/mL

**Table 2:** Time course of level of main blood biochemical parameters.

Laboratory data/day	1	2	3	4	5	6	7	8	9	11	13
Amylase (IU/L)	42	75	94	360	904	1041	792	620	616	600	606
C reactive protein (mg/dL)	0	0.5	3.5	8.8	16.8	12	7.3	14.3	13.1	6.4	2.4
Hemoglobin (g/dL)	11	10.3	8.4	9.4	9.7	10.2	10.7	10.7	10.8	10.9	12.3
Creatinine (mg/dL)	1	0.94	0.83	0.69	0.61	0.59	0.68	0.67	0.71	0.67	0.8
Alanine transaminase (IU/L)	105	171	115	91	73	62	52	45	47	43	37
